# Emerging Sustainable Bioprocess for the Valorization of Agave Bagasse for Single-Cell Protein Production

**DOI:** 10.3390/foods15061033

**Published:** 2026-03-16

**Authors:** Emiro Leal-Urbina, Elisa Dufoo-Hurtado, Marcela Gaytán-Martínez, Edgar N. Tec-Caamal, Aurea K. Ramírez-Jiménez

**Affiliations:** 1 Tecnologico de Monterrey, School of Engineering and Sciences, Ave. Eugenio Garza Sada 2501, Monterrey 64849, NL, Mexico; emirolealurb@gmail.com (E.L.-U.); elisa.dufoo@tec.mx (E.D.-H.); edgart@tec.mx (E.N.T.-C.); 2 Posgrado de Alimentos, Facultad de Quimica, Universidad Autónoma de Querétaro, Querétaro 76010, QRO, Mexico; marcelagaytanm@yahoo.com.mx

**Keywords:** agave bagasse, alternative protein, valorization, circular economy, enzymatic hydrolysis, submerged fermentation, emerging bioprocess

## Abstract

In this work, a food-compatible bioprocess was evaluated for the production of yeast single-cell protein from mezcal agave bagasse. Bagasse was enzymatically hydrolyzed at 10% (*w*/*v*) solids (pH 4.8, 50 °C, 24 h) using commercial enzymes. The resulting liquid was clarified by activated charcoal adsorption and filtration to obtain a hydrolysate suitable for submerged fermentation. Enzymatic hydrolysis released reducing sugars in the range of 11–17 g/L. *Saccharomyces cerevisiae* was cultivated on the clarified hydrolysate under submerged conditions using both flask-scale and 2 L stirred-tank bioreactor experiments. Trials were performed at flask scale with initial sugars at 8, 17, and 50 g/L, and at 2 L stirred-tank bioreactor scale with initial sugars at 20.68 g/L (R1) and 16.30 (R2) g/L. At the flask scale, final biomass concentrations increased with initial sugar level. Values reached 6.18 ± 0.27, 8.02 ± 0.55, and 9.28 ± 0.10 g/L, while crude protein remained below 10% (3.40 ± 0.15 to 8.69 ± 0.09 g/100 g dry weight). In contrast, bioreactor cultivation resulted in higher protein enrichment, with protein contents over 40% under both oxygen regimes (41.71 ± 0.47 to 45.80 ± 0.43 g/100 g dry weight). Overall, the findings support enzymatic hydrolysis coupled with controlled submerged fermentation as a scalable approach for valorizing agave bagasse into protein-enriched yeast biomass.

## 1. Introduction

Over the past decades, population growth combined with changing dietary patterns has increasingly challenged global food production systems. Current projections indicate that the world population may exceed 10 billion within the next few decades, leading to a substantial increase in global protein demand [1]. Under prevailing dietary trends, this demand is expected to depend largely on conventional animal-based protein sources, which are associated with high environmental costs, including intensive land and water use and significant greenhouse gas emissions [2,3]. Together, these challenges underscore the need to develop alternative protein sources that are nutritionally adequate, environmentally sustainable, and scalable.

Among the proposed alternatives, single-cell proteins (SCP) have gained increasing attention as a sustainable protein source. SCP are derived from microbial biomass, mainly yeasts and bacteria, and offer several advantages over traditional protein sources, including rapid biomass formation, high protein content, and efficient substrate-to-biomass conversion under controlled fermentation conditions [4]. Compared with other emerging protein sources such as plant-based isolates or insect protein, SCP cultivation is typically faster, requires less land, and allows for the production of protein with a high degree of compositional control. In practice, SCP production can also be integrated into circular bioeconomy frameworks by coupling microbial growth with the valorization of low-value organic by-products, contributing to both food security and sustainability goals [5,6]. Lignocellulosic residues are particularly attractive due to their abundance, renewability, and high biotechnological potential [7].

In Mexico, the production of agave-based beverages, particularly mezcal, generates substantial amounts of agave bagasse as a solid by-product. This fibrous residue is rich in structural polysaccharides but is often managed through open-air accumulation, burning, or uncontrolled disposal. Such practices result in soil degradation, leachate formation, odor emissions, and greenhouse gas release [8,9]. Recent life-cycle assessment studies have identified agave bagasse management as one of the main environmental bottlenecks within the mezcal production chain [10].

From a biotechnological perspective, the valorization of agave bagasse presents an opportunity to address these environmental challenges while generating value-added products. Enzymatic hydrolysis is widely regarded as a mild and selective strategy for converting lignocellulosic substrates into fermentable sugar streams. Compared with harsher chemical pretreatments, enzymatic processes reduce the formation of inhibitory compounds and preserve carbohydrate availability for subsequent microbial fermentation [11,12]. Combined with submerged fermentation, the resulting hydrolysates can support microbial growth and protein biosynthesis, enabling the transformation of agro-industrial residues into nutritionally relevant biomass [2].

With this framework, *Saccharomyces cerevisiae* represents a robust and food-grade microorganism for SCP production. This yeast has a long history of use in food and beverage fermentations and is widely recognized for its Generally Recognized as Safe (GRAS) status and well-characterized metabolism. *S. cerevisiae* shows strong adaptability to diverse carbon sources, tolerance to residual inhibitors commonly present in lignocellulosic hydrolysates, and the ability to generate protein-rich biomass under submerged fermentation conditions [3,4]. Submerged fermentation systems allow for the precise control of key operational parameters such as aeration, pH, and temperature, which are essential for achieving reproducible biomass yields and enabling process scale-up.

Despite the growing interest in SCP production from agro-industrial residues, the utilization of agave bagasse as a substrate for yeast-based SCP remains limited, particularly when simple and food-compatible processing routes are considered. In this context, the present study evaluates a straightforward strategy for producing SCP from agave bagasse through enzymatic hydrolysis followed by submerged fermentation with *S. cerevisiae*.

## 2. Materials and Methods

### 2.1. Raw Material Collection and Preparation

Agave bagasse was obtained from a mezcal distillery, “La Cascada,” located in central Mexico, and transported to the laboratory immediately after collection. Upon arrival, the material was stored at −20 °C to prevent microbial degradation. The bagasse was thawed and oven-dried at 55 °C until a stable moisture content below 10% (*w*/*w*). The dried material was then milled and sieved to obtain a uniform particle size below approximately 1.7 mm [13].

### 2.2. Enzymatic Hydrolysis of Agave Bagasse

The dried and milled agave bagasse was suspended in distilled water at 10% total solids on a dry matter basis. The slurry was sterilized by autoclaving (121 °C, 15 psi, 15 min). After cooling, pH was adjusted to 4.8 using 1 M HCl or 1 M NaOH prior to enzyme addition. During hydrolysis, pH was monitored and readjusted as needed to maintain pH near 4.8.

Enzymatic hydrolysis was carried out using commercial enzyme preparations Cellic^®^ CTec2 (cellulase complex) and Cellic^®^ HTec2 (hemicellulase/xylanase activity) (Novozymes A/S, Bagsværd, Denmark). CTec2 was used to hydrolyze cellulose into soluble glucose and cellobiose, while HTec2 was added to assist in hemicellulose depolymerization and to improve cellulose accessibility within the lignocellulosic matrix. The cellulase preparation presented an activity of 120.34 FPU/mL. Enzymes were dosed at 0.174 mL CTec2 per g dry bagasse and 0.0127 mL HTec2 per g dry bagasse, corresponding to a CTec2:HTec2 volumetric ratio of 13.7:1. Based on the enzyme volume added and the dry mass of bagasse, the effective cellulase loading corresponded to approximately 20.94 FPU/g dry biomass, while the hemicellulase preparation was added proportionally according to supplier technical guidelines to support synergistic hydrolysis [14].

Hydrolysis was performed at 50 °C with agitation at 160 rpm for 24 h. After incubation, enzymatic activity was stopped by thermal inactivation at 100 °C for 30 s. The hydrolysate was then cooled to room temperature prior to clarification and further processing.

### 2.3. Hydrolysate Clarification

After enzymatic hydrolysis, the liquor fraction was clarified to remove residual solids and to reduce the presence of soluble compounds that could interfere with yeast growth [11,12]. The clarification strategy was designed to preserve food-compatible processing conditions, only aqueous operations were employed, and no organic solvents or chemical detoxification reagents typically used in lignocellulosic fuel processing were applied.

Briefly, the pH was adjusted to 5, and powdered activated charcoal was added at 2% (*w*/*v*). The suspension was incubated at 45 °C under agitation at 150 rpm for 1 h. The treated hydrolysate was centrifuged at 11,000 rpm and 4 °C for 10 min to remove suspended solids and charcoal particles. The supernatant was subsequently clarified through a sequential filtration using laboratory-grade filter paper, followed by Whatman grade 42, grade 2, and grade 1 filters. A final filtration was carried out using a 0.45 μm nylon membrane to obtain a clear, particle-free hydrolysate. The clarified hydrolysate was stored at 4 °C and used within 3 days for fermentation experiments.

### 2.4. Microorganisms and Inoculum Preparation

A food-grade yeast was selected based on its suitability for SCP production, aerobic growth performance, and reported tolerance to lignocellulosic hydrolysates. A commercial strain of *S. cerevisiae* (Tradipan^®^, Mexico City, Mexico) was used. Stock cultures were maintained on yeast extract–peptone–dextrose (YPD) medium under sterile conditions.

Before fermentation, yeast inocula were prepared by cultivating *S. cerevisiae* in liquid YPD medium (Sigma- Aldrich, St. Louis, MO, USA) under aerobic conditions at controlled temperature and agitation until cells reached the exponential growth phase. Cell concentration was determined by direct counting using a Neubauer hemocytometer (ISOLAB Laborgeräte GmbH, Eschau, Germany)under optical microscopy, and optical density at 600 nm (OD_600_) was used as a complementary indicator to adjust inoculum density. To relate OD_600_ to biomass concentration during time-course plots, OD_600_ was converted to dry cell weight (DCW) using a literature calibration for *S. cerevisiae*, where approximately 30 OD units correspond to 7.5 g dry biomass/L, equivalent to a conversion factor of 0.25 g DCW/L per OD_600_, determined gravimetrically and correlated to optical density over a defined linear range [15].

An appropriate volume of the actively growing culture was directly transferred to the fermentation medium to obtain an initial concentration of approximately 1 × 10^7^ cells/mL. The inoculum corresponds to approximately 5–10% (*v*/*v*), depending on the cell density of the pre-culture.

### 2.5. Fermentation Medium and Submerged Fermentation

Clarified agave bagasse hydrolysate was used as the main carbon source for submerged fermentations. To support aerobic growth and protein formation, the hydrolysate was supplemented with food grade nutrients (Sigma- Aldrich, St. Louis, MO, USA) grouped by function: a nitrogen blend, buffering salts, a magnesium source, and a trace mineral solution. The supplementation strategy used in this study was as follows per L of working volume: total added organic nitrogen equivalent of 0.3 to 1.2 g N, total phosphate buffer capacity of 10 to 50 mM, magnesium source equivalent to 0.1 to 1.0 g MgSO_4_, and trace minerals added as 0.1 to 1.0 mL of a food grade premix. Hydrolysate and heat stable salts were autoclaved together. Initial C:N was calculated on a mass basis using DNS-reducing sugars at inoculation expressed as glucose equivalents and total added nitrogen. Across yeast conditions, initial C:N ranged from 12 to 28 [16,17].

Initial reducing sugar concentrations were adjusted according to the experimental conditions and cultivation scale. Enzymatic hydrolysis of agave bagasse releases approximately 17 g/L reducing sugars after 24 h, which serves as the base hydrolysate for fermentation media and was chosen as the baseline condition because it reflects the expected performance of the process without external carbon supplementation. At flask scale, the low (LF, 8 g/L) was prepared from the clarified hydrolysate, medium (MF, 17 g/L), and high (HF, 50 g/L) were obtained by supplementing the hydrolysate with glucose. At the bioreactor scale, two substrate concentrations were evaluated (R1 and R2). Reactor 2 (R2) used only the clarified hydrolysate without external carbon supplementation, while Reactor 1 (R1) consisted of hydrolysate supplemented with glucose to reach the desired concentration. Reported values correspond to reducing sugar measured at inoculation using the DNS method (20.68 g/L for R1 and 16.30 g/L for R2). The initial pH of all fermentation media was adjusted to 5.3 before sterilization. Media were sterilized by autoclaving at 121 °C and 15 psi for 15 min and allowed to cool before inoculation.

Submerged fermentations were conducted at both flask and laboratory bioreactor scales to assess the effects of cultivation scale and oxygen availability on yeast growth and protein production. Samples were collected at defined time points for biomass, protein, and substrate analysis. At flask scale, samples were collected every 1–3 h, while in bioreactor experiments, sampling was more frequent during exponential growth and less frequent during later stages of cultivation.

#### 2.5.1. Flask-Scale Fermentation Conditions

Flask fermentations were carried out in 250 mL Erlenmeyer flasks containing 50 mL of culture medium. Cultures were incubated at 30 °C with orbital agitation at 150 rpm. Substrate availability was defined on the initial reducing sugar concentration determined by the DNS method [17]. During fermentation, pH was not actively controlled; flasks were closed with cotton plugs to permit passive gas exchange.

#### 2.5.2. Bioreactor-Scale Fermentation Conditions

Bioreactor fermentations were performed in stirred-tank systems with a total volume of 2 L and a working volume of 1 L, equipped with online monitoring of pH, temperature, and dissolved oxygen (DO) (BioFlo^®^/CelliGen^®^ 115 Benchtop Fermentor & Bioreactor, Eppendorf, Hamburg, Germany). Cultures were operated in batch mode at 30 °C. Aeration was initially set at 1 vvm, and agitation was provided by mechanical stirring, with impeller speed adjusted to 200–300 rpm to ensure adequate mixing and oxygen transfer. DO was monitored digitally and controlled by setpoint using a cascade strategy. The controller prioritized agitation as the first manipulated variable, increasing impeller speed to meet the DO setpoint. When agitation reached the maximum operating limit, aeration was used as the second manipulated variable and was increased gradually in stepwise increments until the DO setpoint was recovered and maintained. This control approach allowed for oxygen transfer to be increased without abrupt changes in gas flow while maintaining the same DO setpoint framework used across bioreactor runs.

Scale-up from flask to bioreactor was based primarily on increasing agitation intensity to improve oxygen availability, while keeping medium composition and other physicochemical conditions consistent with flask-scale experiments.

Two reactor conditions (R1 and R2) were used to evaluate the influence of oxygen availability and substrate composition on yeast growth and protein production. In Reactor 1 (R1), dissolved oxygen was actively controlled and maintained above 30% air saturation through adjustment of aeration rate and agitation speed. In Reactor 2 (R2), dissolved oxygen was not strictly maintained above this level during the initial phase of fermentation, allowing for partial oxygen limitation before control was gradually introduced at later stages. Throughout bioreactor experiments, pH was monitored online and actively controlled at 5.3 by automated addition of acid or base. All other operating parameters were kept constant.

### 2.6. Analytical Methods

#### 2.6.1. Biomass Quantification

Yeast biomass was determined by dry cell weight (DCW). Briefly, defined culture volumes were centrifuged at 5000× *g* for 10 min, and the resulting pellets were washed twice with distilled water to remove residual medium components. The biomass was then transferred to pre-weighed containers and dried at 60 °C until constant weight. DCW was determined at the end of fermentation (72 h). Biomass concentration was calculated by subtracting the tare weight and normalizing to the original sample volume, and expressed as grams of dry biomass per liter of culture. In parallel, yeast growth during fermentation was monitored by measuring optical density at 600 nm (OD_600_) using a spectrophotometer.

#### 2.6.2. Protein Determination

Crude protein content of the yeast biomass was determined using the Kjeldahl nitrogen analysis method, following standardized procedures [18], where results are expressed on a dry biomass basis using protein (%) = N (%) × Nitrogen-to-Protein Factor Conversion (6.25 for Yeast and 5.7 for Fungi). Dried biomass samples (approximately 100 mg) were digested with concentrated sulfuric acid in the presence of a potassium sulfate–copper sulfate catalyst to convert organic nitrogen to ammonium. Ammonia was subsequently distilled into a boric acid solution and quantified by titration with standardized 0.1 N hydrochloric acid.

Total nitrogen content was converted to crude protein using a nitrogen-to-protein conversion factor of 6.25. Protein content was expressed as the percentage of protein of dry biomass basis.

#### 2.6.3. Sugar Analysis

Reducing sugars in hydrolysates and fermentation samples were quantified using the 3,5-dinitrosalicylic acid (DNS) colorimetric assay [19]. Calibration curves were prepared using glucose as the reference standard, and absorbance was measured at 540 nm using a spectrophotometer. Results are reported as reducing sugar equivalents (g/L, expressed as glucose equivalents).

Because the DNS assay quantifies total reducing equivalents, it does not distinguish individual monosaccharides from short reducing oligosaccharides or other reducing compounds that may be present in lignocellulosic hydrolysates. Accordingly, all sugar concentrations are reported as DNS reducing sugar equivalents (g/L, glucose equivalents), and the substrate variable S used in yield calculations and kinetic modeling represents this total reducing equivalent pool. DNS values were used as an operational indicator of the overall reducing sugar equivalent pool and its temporal decrease during fermentation, rather than as a specific quantification of individual fermentable sugars. For flask-scale experiments, samples were analyzed every 1–3 h, whereas in bioreactor fermentations, sampling was more frequent during the exponential growth phase and less frequent during later cultivation stages.

### 2.7. Kinetic Modeling and Parameter Estimation

Biomass growth curves (X, g/L) were fitted to four commonly used empirical growth models: Logistic, Gompertz, Richards, and von Bertalanffy (Equations (1)–(4), respectively). Model parameters were estimated by nonlinear least-squares regression using experimental time-series biomass data. Goodness-of-fit was evaluated using the coefficient of determination (*R*^2^) and root mean square error (*RMSE*) (Equations (5) and (6)).

(1) $$\frac{dX}{dt}={µ}_{max}\cdot X(1-\frac{X}{X_{max}})$$

(2) $$X(t)=K\cdot exp\{-exp[-\alpha (t-t_{0})]\}$$

(3) $$X(t)=A+\frac{K-A}{{\left(C+Q\cdot e^{-B(t-M)}\right)}^{\frac{1}{v}}}$$

(4) $$\frac{dX}{dt}=k(X_{\infty }-X)\to X(t)=X_{\infty }(1-e^{-kt})$$

(5) $$R^{2}=1-\frac{\Sigma {\left(y_{obs}-y_{pred}\right)}^{2}}{\Sigma {\left(y_{obs}-{\underline{y}}_{obs}\right)}^{2}}$$

(6) $$RMSE=\sqrt{\frac{1}{n}\Sigma {\left(y_{obs}-y_{pred}\right)}^{2}}$$

Model performance was evaluated using the highest *R*^2^ and lowest *RMSE*. From the selected fits, the maximum specific growth rate (µ*_max_*, 1/h) and the maximum biomass concentration (*X_max_*, g/L) were determined. Biomass yield on substrate (*Y_X/S_*, gX/gS) was calculated from experimental biomass formation and reducing sugar consumption profiles, using reducing sugar concentrations measured by the DNS method and expressed as glucose equivalents.

### 2.8. Statistical Analysis

All fermentation experiments were performed in duplicate, and results are reported as mean values ± standard deviation (*n* = 2). Differences among multiple experimental conditions, including low, medium, and high levels, were evaluated using one-way analysis of variance (ANOVA). When statistically significant differences were detected, Tukey’s HSD post hoc test was applied.

Bioreactor experiments were conducted in two independent runs (R1 and R2). Differences between reactor conditions were assessed using Student’s *t*-test by comparing final biomass concentration, crude protein content, and residual reducing sugar levels between R1 and R2 fermentations. Statistical significance was defined at *p* < 0.05. All statistical analyses were performed using JMP software (version 10; SAS Institute Inc., Cary, NC, USA).

## 3. Results

### 3.1. Enzymatic Hydrolysis and Reducing Sugar Availability

Enzymatic hydrolysis of mezcal agave bagasse led to the release of soluble reducing sugars suitable for yeast cultivation. Under the selected conditions (10% *w*/*v* solids, pH 4.8, 50 °C, and a measured cellulolytic activity of 120.34 FPU/mL), the clarified hydrolysates consistently contained reducing sugar concentrations in the range of 11–17 g/L.

Clarification caused a small loss of soluble sugars. Reducing sugars (expressed as glucose equivalents) decreased from 18.28 ± 0.11 g/L before clarification to 16.99 ± 0.33 g/L after clarification, corresponding to a reduction of 7.05 ± 2.31%. In the batch processed at 600 mL, reducing sugars decreased from 18.28 ± 0.11 g/L to 17.09 ± 0.10 g/L, corresponding to a reduction of 6.49 ± 1.08%. These results show that the clarification step removed less than 10% of the soluble sugar fraction while conditioning the hydrolysate for fermentation.

At an early stage (t = 10 h), reducing sugar concentrations reached 9.37 ± 3.20 g/L. Prolonging the reaction to 96 h resulted in a moderate increase to 12.33 ± 4.14 g/L, corresponding to an absolute gain of approximately 3 g/L of reducing sugars.

Based on this behavior, a hydrolysis time of 24 h was selected for all subsequent fermentation experiments, as it provides a representative soluble carbon concentration while avoiding unnecessarily long processing time. To evaluate yeast performance under increasing carbon availability, three substrate levels were defined for fermentation assays. Low and medium substrate levels were derived exclusively from the sugars released during agave bagasse hydrolysis, whereas the high substrate level was obtained by supplementing the hydrolysate with glucose as an additional readily fermentable carbon source. In all cases, the agave bagasse hydrolysate remained the main substrate matrix for fermentation.

### 3.2. Flask-Scale Fermentation Outcomes

Flask-scale fermentations were carried out to assess the growth behavior of *S. cerevisiae* on agave bagasse hydrolysates under different initial reducing sugar concentrations. Three substrate availability levels were evaluated: low (8 g/L), medium (17 g/L), and high (50 g/L). Biomass formation and reducing sugar consumption were monitored over a total fermentation time of 72 h.

**Figure 1:**
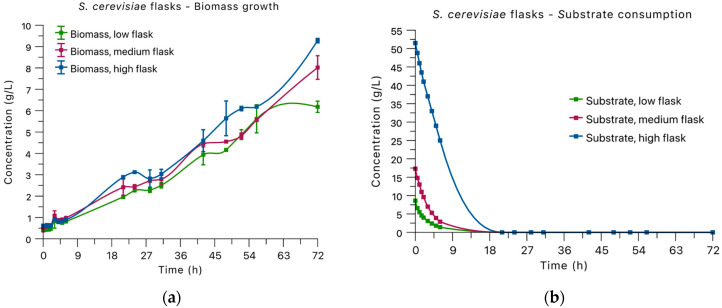
Biomass growth and reducing sugar consumption by *S. cerevisiae* during flask-fermentation of agave bagasse hydrolysates under different initial sugar concentrations: (**a**) Biomass concentrations. (**b**) Substrate consumption.

Across all substrate levels, reducing sugar concentrations decreased rapidly during the early stages of fermentation. In each treatment, most reducing sugars were consumed within 18–24 h (Figure 1b). After this consumption phase, residual reducing sugar concentrations remained low, suggesting near-complete utilization of available sugars from the hydrolysate. Despite this apparent depletion, biomass continued to increase for several hours. One possible explanation is the utilization of carbohydrates not fully detected by the DNS assay, such as short-chain oligosaccharides commonly present in lignocellulosic hydrolysates. In addition, *S. cerevisiae* can rely on intracellular reserve carbohydrates, including glycogen and trehalose, which are known to support cellular maintenance and limited biomass formation under carbon-limited conditions [20]. Under aerobic conditions, yeast may also reassimilate previously produced fermentation metabolites such as ethanol once readily available sugars are depleted [21]. Because the DNS method quantifies total reducing equivalents in the culture medium rather than individual sugars or intracellular carbohydrate pools, a more detailed carbohydrate and metabolites characterization would help clarify carbon utilization dynamics in agave bagasse hydrolysates and will be considered in future studies.

Yeast biomass concentration increased with increasing initial sugar availability. Final dry biomass concentrations at 72 h were 6.18 ± 0.27 g/L in low-sugar flasks, 8.02 ± 0.55 g/L in medium-sugar flasks, and 9.28 ± 0.10 g/L in high-sugar flasks (Table 1). In all cases, biomass accumulation continued after reducing sugars approached residual levels, and growth curves displayed a multiphasic pattern during fermentation (Figure 1a). Although biomass stabilized after approximately 27–34 h, fermentations were monitored up to 72 h to confirm the establishment and maintenance of the stationary phase.

The relationship between initial reducing sugar concentration and final biomass was not proportional across treatments. Although higher carbon availability resulted in increased biomass formation, the relative gain in biomass diminished at higher sugar levels, particularly between the medium and high substrate conditions. This behavior is partly associated with differences in substrate composition since the high condition contained supplemented glucose while the low and medium conditions were primarily derived from hydrolysate sugars. Because *S. cerevisiae* preferentially metabolizes glucose, the observed biomass increase reflects both carbon availability and the presence of a readily assimilable carbon source rather than concentration alone.

Protein content of the harvested yeast biomass also increased with substrate availability. Crude protein content reached 3.40 ± 0.15 g protein/100 g dry biomass at low sugar levels, 5.66 ± 0.39 g protein/100 g dry biomass at medium levels, and 8.69 ± 0.09 g protein/100 g dry biomass under high sugar conditions (Table 1). Similar to biomass formation, higher initial sugar concentrations supported greater protein accumulation in the final biomass.

Overall, flask-scale fermentations showed that enzymatically hydrolyzed agave bagasse can sustain active yeast growth across a wide range of initial sugar concentrations. Increasing substrate availability enhanced both biomass formation and protein content, although diminishing returns were observed at the highest sugar levels.

### 3.3. Bioreactor-Scale Fermentation Outcomes

**Figure 2:**
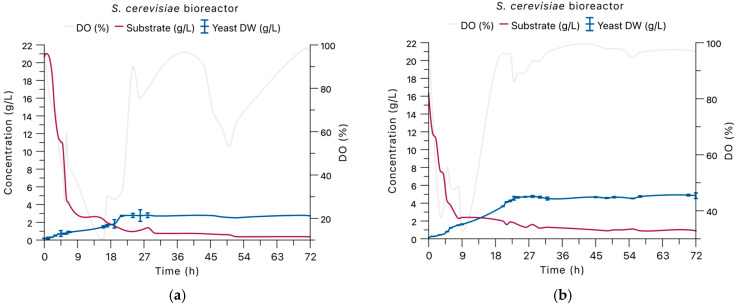
Time-course profiles of dissolved oxygen, substrate consumption, and biomass formation during bioreactor-scale fermentation of *S. cerevisiae* grown on agave bagasse hydrolysates under different oxygen regimes: (**a**) Oxygen-sufficient conditions (R1). (**b**) Dynamic Oxygen regime (R2).

Bioreactor-scale fermentations were performed to evaluate the influence of oxygen regime and substrate utilization on yeast growth during cultivation on agave bagasse hydrolysates. Two reactor configurations were evaluated: Reactor 1 (R1), operated under oxygen-sufficient conditions with dissolved oxygen actively maintained above 30% air saturation, and Reactor 2 (R2), operated under a dynamic oxygen regime in which oxygen depletion was initially allowed before control was gradually reintroduced. Temporal profiles of dissolved oxygen, reducing sugar concentration, and biomass formation under both reactor conditions are presented in Figure 2.

In both reactor configurations, reducing sugar was depleted rapidly, with near-complete consumption occurring within the first 18 h of fermentation. Although substrate utilization followed similar kinetics in both cases, clear differences were observed in growth behavior and final biomass accumulation between reactor conditions.

In reactor 1 (R1), operated under oxygen-sufficient conditions with DO maintained above 30% air saturation, *S. cerevisiae* displayed a single exponential growth phase followed by an early transition to the stationary phase. Under these conditions, the final biomass concentration reached 2.72 ± 0.01 g/L, while crude protein was 41.71 ± 0.47 g protein/100 g dry biomass (Table 2).

In contrast, Reactor 2 (R2), which was operated under a dynamic oxygen regime allowing for initial oxygen depletion before re-establishing DO control, showed a different growth response. Biomass concentration increased to 4.85 ± 0.30 g/L, corresponding to an approximate 78% increase relative to R1, despite the lower initial reducing sugar concentration. Protein content in R2 biomass reached 45.80 ± 0.43 g protein/100 g dry biomass, exceeding the values obtained under continuously oxygen-sufficient conditions.

Notably, the use of non-supplemented hydrolysate in R2 (16 g/L reducing sugars) confirmed that reducing sugars released exclusively through enzymatic hydrolysis were sufficient to sustain yeast growth and protein accumulation at the bioreactor scale. Compared with flask-scale experiments, bioreactor cultivation consistently yielded higher protein fractions, exceeding 40% (dry weight basis) under both oxygen regimes.

Taken together, these results indicate that oxygen management, rather than substrate availability alone, played a central role in shaping biomass formation and protein enrichment in *S. cerevisiae* during bioreactor-scale fermentation.

### 3.4. Gowth Kinetics and Model Outputs

**Figure 3:**
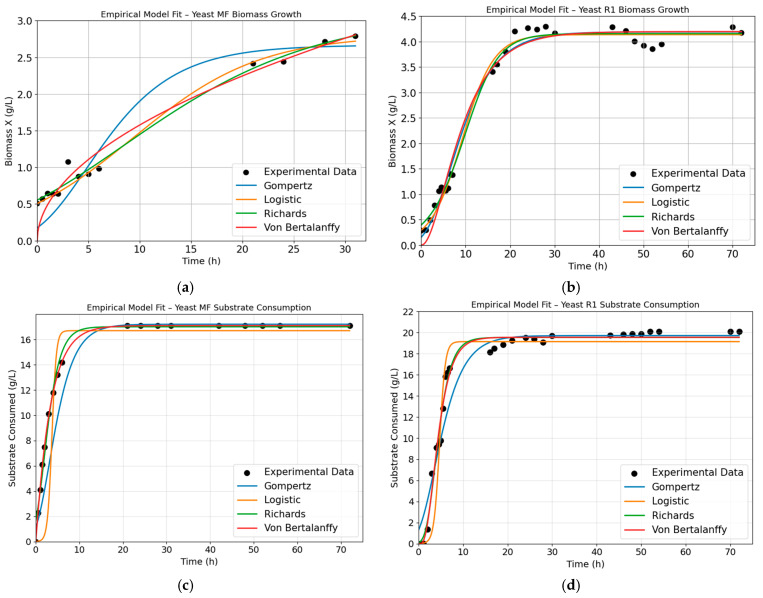
Visual comparison of empirical model fits for *S. cerevisiae* in representative conditions MF (flask) and R1 (2 L bioreactor). Black markers show experimental measurements. Colored curves show nonlinear regressions using the four empirical models: Gompertz in blue, Logistic in yellow, Richards in green, and von Bertalanffy in red. Panels (**a**,**b**) show biomass growth fits for MF and R1, respectively. Panels (**c**,**d**) show substrate consumption fits for MF and R1, respectively.

To compare yeast performance across all scales, time-series data for biomass (*X*, g/L) and soluble reducing sugars (Figure 3c,d) (*S*, g/L as glucose equivalents by DNS) were modeled for the five experimental datasets: LF, MF, HF (flasks), and R1, R2 (2 L bioreactors). Four empirical growth models were evaluated for *X*(t) using Equations (1)–(4), respectively (Logistic, Gompertz, Richards, and von Bertalanffy). For *S*(t), empirical depletion functions were tested to describe sugar consumption dynamics under each condition.

Model screening was first performed using two representative datasets, MF (flask, intermediate sugar) and R1 (bioreactor) (Figure 3), to establish initial parameter guesses and to identify the most suitable model form for each response variable. For each candidate model, parameters were estimated by nonlinear least-squares regression. Fits were ranked using *RMSE* and *R*^2^. This initial screening step showed that the Richards model best described *S. cerevisiae* biomass growth, capturing the asymmetric sigmoidal rise and the transition toward a stationary plateau under both flask and reactor conditions. For substrate consumption, the von Bertalanffy model provided the best fit, describing the progressive deceleration in depletion as sugars are fully consumed.

After selecting the best-performing model per variable from MF and R1, the same model choices were applied to the remaining datasets (LF, HF, and R2) to keep parameter comparability across all tested conditions. Final fits remained strong across all yeast runs (R2 from 0.96 to 0.99 for biomass, and 0.99 to 1.00 for reducing sugar depletion), supporting the use of Richards for *X*(t) (Table 3) and von Bertalanffy for *S*(t) (Table 4) as a consistent kinetic description of yeast growth and sugar consumption on agave bagasse hydrolysate. The resulting kinetic parameters are summarized below.

Because reducing sugars were quantified by the DNS method, S represents total reducing equivalents rather than individual fermentable monosaccharides. Therefore, kinetic parameters, including the apparent biomass yield on substrate (*Y_X/S_*), should be interpreted comparatively between operational conditions rather than as absolute stoichiometric conversion efficiencies. Under aerobic, biomass-oriented cultivation of *S. cerevisiae*, biomass yields on glucose are commonly reported on the order of ~0.25–0.55 gX/gS depending on oxygen availability and nutrient balance, with values approaching ~0.5 gX/gS under well-aerated, respiration-oriented conditions [21,22]. In this context, the apparent yield obtained under oxygen-controlled operation (*Y_X/S_* ≈ 0.30 g/g, calculated from DNS glucose equivalents) is consistent with literature expectations. In contrast, the lower yields estimated in shake flasks are consistent with reduced oxygen transfer capacity and less efficient carbon allocation toward biomass formation under oxygen-limited conditions [21].

## 4. Discussion

The enzymatic hydrolysis of mezcal agave bagasse produced reducing sugar concentrations within the commonly reported range for non-pretreated or mildly treated agave residues. Values between 11 and 17 g/L highlight the intrinsic recalcitrance of agave lignocellulose, while confirming that standalone enzymatic approaches can release enough reducing sugars to sustain yeast growth without the need for harsher or more aggressive physicochemical pretreatments. Similar sugar concentrations have been reported for *Agave tequilana* bagasse, with total sugar concentrations of approximately 12–15 g/L under optimized enzymatic conditions [23].

In the present study, the use of relatively high cellulolytic activity allowed for the effective saccharification of agave bagasse without chemical pretreatment, supporting the feasibility of mild, food-compatible enzymatic strategies for residue valorization. Extending hydrolysis beyond 20–24 h resulted in only limited additional sugar release, indicating that saccharification approaches a quasi-steady state. This behavior is consistent with previous observations in lignocellulosic systems and is commonly attributed to reduced enzyme accessibility, substrate recalcitrance, product inhibition, and mass transfer limitations [24,25]. From a process standpoint, selecting a 24 h hydrolysis time represents a reasonable compromise between sugar recovery and overall process efficiency, particularly for SCP-oriented applications where moderate sugar concentrations are sufficient.

Flask-scale fermentations showed that *S. cerevisiae* efficiently utilized reducing sugars derived from the agave bagasse hydrolysate across a wide range of initial substrate concentrations. In all treatments, most of the available sugars were consumed within 18–24 h, indicating a high substrate uptake capacity even when hydrolysate-derived sugars were the primary carbon source. Despite similar substrate consumption profiles, yeast did not follow a classical biphasic diauxic pattern, but instead displayed multiphasic behavior. Such growth profiles have been reported for yeasts cultivated on complex substrates containing mixed sugars and short-chain oligosaccharides, where sequential substrate utilization and metabolic reprogramming can overlap [20,21]. The continued increase in biomass after apparent sugar depletion suggests the involvement of secondary carbon sources or the mobilization of intracellular storage carbohydrates, such as glycogen and trehalose, which are known to support late-stage growth under carbon-rich or transition conditions.

At higher initial sugar concentrations, biomass yields increased only modestly relative to the amount of substrate supplied, suggesting a decrease in carbon-to-biomass conversion efficiency. This behavior is commonly associated in yeast systems with overflow-type metabolism or Crabtree-like responses, in which high sugar availability can shift carbon utilization patterns even under aerobic conditions. In the present work, fermentative by-products were not directly quantified; therefore, this interpretation should be considered indicative rather than conclusive. Nevertheless, the combination of rapid sugar consumption, multiphasic growth, and low protein fraction in shake-flask is consistent with conditions where oxygen transfer becomes limiting relative to carbon availability. Under these conditions, protein accumulation remained below 10% dry weight basis. Together, these observations point to inherent limitations of flask-scale systems for assessing SCP productivity and underscore the importance of adequate aeration when targeting protein enrichment in yeast-based processes.

Similar behavior has been reported in yeast SCP production on lignocellulosic hydrolysates, where shake-flask cultivation frequently results in low protein fractions due to oxygen transfer limitations. Under oxygen limitation, *S. cerevisiae* often shifts toward fermentative metabolism with reduced protein accumulation, and protein yields below 10–15% dry weight have been reported for oxygen-limited flask cultures on lignocellulosic substrates [21]. Studies using agricultural residues such as wheat straw hydrolysate and corn stover hydrolysate have shown that protein content in *S. cerevisiae* biomass can remain below 15% dry weight in poorly aerated systems but increases substantially under controlled aeration in bioreactors. These observations support the interpretation that the low protein fraction observed in the present flask experiments is primarily related to oxygen availability rather than substrate inadequacy.

The role of oxygen availability became more apparent at the bioreactor scale. Under oxygen-sufficient conditions, *S. cerevisiae* exhibited a single exponential growth phase followed by early stabilization of biomass concentration, accompanied by substantially higher protein content (>40% dry weight). In contrast, controlled aeration in stirred bioreactors stabilizes metabolism and increases protein fractions to values consistent with industrial SCP benchmarks, commonly in the 40–60% range for yeast biomass, aligning with the 41–46% obtained in this study [2,6,26]. The higher protein density under regulated oxygen supply is consistent with improved respiratory metabolism and amino acid synthesis efficiency in aerobic yeast cultivation [15].

Although metabolic fluxes were not directly measured, the higher protein fractions and improved growth kinetics observed under controlled aeration are consistent with a metabolism oriented toward biomass synthesis rather than by-product formation. These observations agree with previous reports indicating that adequate oxygen availability in yeast cultures favors biosynthetic activity and increases protein enrichment in SCP-oriented processes [15].

In contrast, bioreactor operation under a dynamic oxygen regime, with transient oxygen limitation followed by reoxygenation, resulted in increased biomass accumulation and a slight additional increase in protein content. Such responses are consistent with the known physiological flexibility of yeast under changing oxygen conditions, where transient oxygen limitation followed by re-aeration can alter growth patterns and biomass accumulation [27]. These findings further emphasize the importance of oxygen management as a key lever for balancing biomass formation and protein quality in yeast-based SCP production.

The large differences in protein content between flask- and bioreactor-scale results can be explained by the distinct oxygen transfer regimes and by differences in biomass recovery. Flask cultures were oxygen-transfer limited and were cultivated in hydrolysate that may contain fine insoluble residues [28,29]. Biomass was recovered by centrifugation and washed twice with distilled water prior to drying; however, under flask conditions, non-cell solids can still co-sediment with cells despite washing. This would dilute nitrogen on a dry weight basis and lower the apparent crude protein fraction. In bioreactors, stronger mixing and aeration improved oxygen availability and likely reduced co-sedimentation effects, yielding crude protein values that are more typical of yeast SCP [30,31,32]. Future work will evaluate fed-batch strategies to maintain controlled carbon availability while minimizing high initial sugar loads and their potential impacts on yeast physiology [33].

The kinetic analysis further supports oxygen availability as a key driver of carbon conversion by *S. cerevisiae* grown on agave bagasse hydrolysate. An initial screening using the MF flask condition and the R1 condition was performed to select appropriate model structures based on RMSE and R^2^ values. For the biomass growth, the Richards model consistently provided the best description, capturing both the asymmetric growth phase and the gradual deceleration toward a stationary plateau more effectively than the Logistic and Gompertz models. This behavior is consistent with previous reports highlighting Richard’s model’s greater flexibility in describing microbial growth under variable environmental conditions [34,35].

For substrate consumption, the von Bertalanffy model was used because it accurately described the progressive slowing of sugar depletion as concentrations approached exhaustion. Similar von Bertalanffy-type formulations have been successfully applied to biological consumption trends characterized by decelerating kinetics [36,37]. Once the model structures were selected, they were applied consistently across all yeast datasets to allow for the direct comparison of kinetic parameters. The fitted parameters revealed clear effects of cultivation scale and oxygen availability. In shake-flask fermentations, maximum specific growth rates (µ_max_) remained low from 0.13 to 0.15 1/h, and biomass yields on substrate Y_X/S_ decreased markedly at the highest sugar condition (HF, 0.05 g/g). In contrast, operation under oxygen-controlled bioreactor conditions (R2) increased µ_max_ to 0.39 1/h and improved Y_X/S_ to 0.30 g/g. These trends quantitatively support the experimental observations: flask cultures were likely constrained by oxygen transfer, whereas improved oxygen control in the bioreactor coincided with higher growth rates, improved substrate utilization efficiency, and increased protein accumulation. While metabolic pathways were not directly analyzed, the kinetic parameters are consistent with a shift toward biomass-oriented growth under controlled aeration.

Comparison between flask-scale and bioreactor-scale fermentation therefore reinforces oxygen availability as a primary determinant of SCP quality in yeast. While shake-flask cultures were dominated by oxygen-limited metabolism and low protein density, bioreactor cultivation stabilized yeast metabolism and enabled protein contents comparable to those reported for industrial yeast-based SCP production [2,26]. Importantly, the successful use of non-supplemented hydrolysate at bioreactor scale confirms that sugars released exclusively through enzymatic hydrolysis of agave bagasse are sufficient to sustain yeast growth and high protein accumulation, supporting the feasibility of this residue as a standalone substrate for SCP production.

A relevant consideration for process translation beyond laboratory scale is the economic and operational impact of the hydrolysis and clarification steps. Enzymatic saccharification is widely recognized as one of the principal cost contributors in lignocellulosic bioprocesses [38]. However, in the present work complete carbohydrate conversion was not required, as moderate concentrations of reducing sugars were sufficient to sustain yeast growth and protein enrichment. This suggests that enzyme loading could be optimized toward partial hydrolysis rather than maximum sugar release, which may substantially improve process economics [39]. In addition, industrial enzyme preparations are already widely used in food and fermentation industries, and their cost has progressively decreased as production capacity has expanded [22]. Although glucose supplementation was used in selected conditions to establish defined carbon availability for comparative purposes, reliance on commercial sugars would likely increase operating costs and reduce the circular valorization advantage of using agave bagasse as the primary carbon source. In this regard, the R2 condition—operated with non-supplemented hydrolysate—highlights a practical advantage for scale-up scenarios aiming to minimize external carbon inputs.

Regarding hydrolysate conditioning, activated charcoal was used at laboratory scale to reduce soluble inhibitory compounds and improve culture reproducibility [12]. Although single-batch charcoal detoxification may not represent the final industrial configuration, similar conditioning strategies are routinely implemented using continuous adsorption systems, membrane clarification, or integrated solid–liquid separation operations in fermentation facilities [11]. Therefore, the charcoal treatment applied here should be interpreted as a proof-of-concept conditioning step demonstrating that inhibitor levels can be reduced sufficiently to enable yeast cultivation. In a scaled process, this operation would likely be integrated with upstream solid separation and medium conditioning steps, minimizing additional processing costs.

Overall, the results indicate that enzymatically hydrolyzed mezcal agave bagasse can serve as a viable carbon source for yeast-based SCP production. Process performance, however, was strongly influenced by oxygen availability, particularly at larger scales. While shake-flask experiments provided useful preliminary information on substrate utilization and growth trends, they were inherently limited to oxygen transfer constraints. In contrast, bioreactor operation with controlled oxygen regimes proved essential to achieve protein enrichment levels comparable to industrial SCP benchmarks. From a process development perspective, these findings highlight oxygen management as a central design variable for yeast-based SCP systems using lignocellulosic hydrolysates. Further work should therefore focus on refining oxygen transfer strategies, optimizing nitrogen availability, and tailoring hydrolysate composition to improve protein productivity and operational robustness. In parallel, consideration of downstream processing and techno-economic performance will be necessary to support the transition of agave bagasse–based SCP production toward pilot and industrial scales within circular bioeconomy frameworks. A limitation of this study is that fermentation by-products such as ethanol or organic acids were not measured. Future work including metabolite profiling will be necessary to confirm the metabolic mechanisms underlying the observed oxygen-dependent behavior.

## 5. Conclusions

This study demonstrates the feasibility of converting mezcal agave bagasse into yeast-based SCP through enzymatic hydrolysis followed by submerged fermentation. Enzymatic saccharification generated fermentable hydrolysates capable of supporting *Saccharomyces cerevisiae* growth, confirming the suitability of this agro-industrial residue as a food-compatible carbon source. At the flask scale, yeast efficiently consumed hydrolysate-derived sugars; however, oxygen transfer limitations led to multiphasic growth behavior and low protein accumulation.

In contrast, bioreactor cultivation under controlled oxygen regimes substantially enhanced protein enrichment, yielding yeast biomass with protein content exceeding 40% on a dry weight basis, and identifying oxygen availability as a key determinant of SCP quality. Importantly, the successful use of non-supplemented hydrolysates at bioreactor scale showed that sugars released exclusively through enzymatic hydrolysis were sufficient to sustain yeast growth and high protein accumulation, reinforcing the industrial relevance of the proposed process. Together, these results support enzymatic hydrolysis coupled with submerged fermentation as a promising route for valorizing agave bagasse into microbial protein. Future studies should address process optimization, downstream processing, and safety and functionality assessments to advance this approach toward industrial implementation.

## Figures and Tables

**Table 1:** Biomass production and protein content of *S. cerevisiae* during flask-scale fermentation of agave bagasse hydrolysates under different substrate availability levels.

Treatment	Substrate (g/L)	Biomass (g/L)	Protein (g Protein/100 g DW)
LF	8.60	6.18 ± 0.27 ^B^	3.40 ± 0.15 ^C^
MF	17.30	8.02 ± 0.55 ^A^	5.66 ± 0.39 ^B^
HF	51.50	9.28 ± 0.10 ^A^	8.69 ± 0.09 ^A^

LF: Low-sugar flask; MF: Medium-sugar flask; HF: High-sugar flask. Different uppercase letter superscripts indicate significant differences among treatments according to Tukey’s HSD test (*p* < 0.05). Values are expressed as mean ± standard deviation (*n* = 2).

**Table 2:** Biomass production and protein content of *S. cerevisiae* during bioreactor-scale fermentation of agave bagasse hydrolysate.

Treatment	Substrate (g/L)	Biomass (g/L)	Protein (g Protein/100 g DW)
R1	20.68	2.72 ± 0.01	41.71 ± 0.47
R2	16.30	4.85 ± 0.30 *	45.80 ± 0.43 *

R1: Reactor 1 operated under oxygen-sufficient conditions with dissolved oxygen maintained above 30% air saturation; R2: Reactor 2 operated under a dynamic oxygen regime, allowing for initial oxygen depletion followed by later control of aeration and agitation; * indicate significant differences between reactor operating conditions (R1 vs. R2) according to Student’s *t*-test (*p* < 0.05). Values are expressed as mean ± standard deviation (*n* = 2).

**Table 3:** Kinetic parameters obtained from the Richards model for *S. cerevisiae*, describing biomass growth across flask (LF, MF, HF) and reactor (R1, R2) scales. Reported parameters include specific growth rate (µ*_max_*), maximum biomass concentration (*X_max_*), biomass yield on substrate (*Y_x/s_*), maximum growth rate (*dX/dt max*), rate constant (*k*), root mean square error (*RMSE*), and coefficient of determination (*R*^2^).

Dataset	µ*_max_* (h^−1^)	*X_max_* (g/L)	*Y_X/S_* (gX/gS)	*dX/dt Max* (g·L^−1^·h^−1^)	*k* (1/h)	*RMSE*	*R* ^2^
LF	0.15	2.90	0.24	0.08	0.08	0.08	0.98
MF	0.14	3.13	0.13	0.09	0.09	0.09	0.99
HF	0.13	3.08	0.05	0.12	0.23	0.12	0.99
R1	0.14	2.73	0.13	0.11	0.20	0.19	0.96
R2	0.39	4.74	0.30	0.26	0.19	0.15	0.99

**Table 4:** Kinetic parameters obtained from the Von Bertalanffy model fit for substrate consumption across flask (LF, MF, HF) and reactor (R1, R2) scales. Parameters include maximum substrate consumed (*S_max_*), maximum substrate consumption rate (*dS/dt max*), rate constant (*k*), root mean square error (*RMSE*), and coefficient of determination (*R*^2^).

Dataset	*S_max_* (g/L)	*dS/dt Max* (g·L^−1^·h^−1^)	*k* (1/h)	*RMSE*	*R* ^2^
LF	8.61	4.11	0.24	0.03	1.00
MF	17.31	4.78	0.30	0.05	1.00
HF	52.12	5.21	0.15	0.85	1.00
R1	19.76	3.46	0.45	0.79	0.99
R2	15.04	3.29	0.31	0.47	0.99

## Data Availability

The original contributions presented in this study are included in the article. Further inquiries can be directed to the corresponding author.

## Abbreviations

The following abbreviations are used in this manuscript:

SCP	Single-cell protein
GRAS	Generally Recognized as Safe
YPD	Yeast Extract Peptone Dextrose
DNS	Dinitrosalicylic Acid
DO	Dissolved Oxygen
DCW	Dry Cell Weight
ANOVA	Analysis of Variance
FPU	Filter Paper Unit
